# Chloroform—Its Method of Administration, Its Dangers and Their Treatment

**Published:** 1896-04

**Authors:** John Parmenter

**Affiliations:** Buffalo, N. Y., Professor of anatomy and adjunct professor of surgery in the Medical Department, University of Buffalo. 519 Franklin Street


### CHLOROFORM—ITS METHOD OF ADMINISTRATION, ITS DANGERS AND THEIR TREATMENT.

By JOHN PARMENTER, M. D., Buffalo, N. Y.,
Professor of anatomy and adjunct professor of surgery in the Medical Department, University of Buffalo.

THE writer begs that the members of the Academy will pardon him for having chosen so hackneyed a theme for this evening's discussion. It is his experience, however, that chloroform is very commonly improperly administered, that its signs of danger are often misunderstood or ignored, and that when death is threatening, only too often the best means of averting it are either omitted or ineffectually employed. It would seem, therefore, not inappropriate to regard this subject as sufficiently practical and important to claim our attention even though we of necessity go over old ground. It is along the lines mentioned that the subject will be presented for your kindly consideration. I wish, further, to say that I shall not enter upon a discussion of the question,

1. Read at the Buffalo Academy of Medicine before the Section on Surgery.

chloroform *vs.* ether, for I think we are all pretty well agreed that chloroform has something of the same relation to ether that morphia has to opium. It is more powerful, it is given in smaller doses, and yet has advantages which make it too valuable to be discarded. It is our duty, then, to learn how to use it properly, thus minimising its dangers and more effectually combatting them should they arise.

*Physical Properties.*—Pure chloroform is a clear, colorless, very volatile liquid. It has a pleasant aromatic odor, which disappears entirely upon complete evaporation. It has a sweetish taste.

*Quality.*—How often do we give a thought as to the purity of the chloroform we use? Yet this is a most important consideration. Among the many impurities found in chloroform the most common are adulterations with spirits of wine, ether, and the like, the very dangerous compounds of methyl formed during its manufacture, and in the decomposition products which develop when the drug is long exposed to air and light. These are free chlorine, compounds of the hydrocarbons with chlorine, aldehyde, hydrochloric acid, acetic acid and formic acid. How, then, may we know the impure from the pure drug? The simplest and most practical test is Hepp's smelling test. This is made by dipping chemically pure filter paper (the so-called Swedish filter paper,) into the chloroform and allowing the latter to evaporate. Pure chloroform leaves no odor. If the chloroform is impure, a more or less sharp, irritating and unpleasant smell remains. I have seen most persistent and dangerous vomiting, headache and stupor, lasting several hours, occur from the use of chloroform afterward found to contain a large quantity of methyl compounds. The conclusions to be drawn from such experience are, that only chemically pure chloroform should ever be used and that pure chloroform should be bought in quantities proportionate to the demand of the physician for it and kept in dark-colored, well-stoppered bottles. The addition of $\frac{1}{2}$ to 1 per cent. of absolute alcohol prevents decomposition and in no way diminishes the value of the chloroform as an anesthetic.

*Administration of Chloroform.*—Under this heading we may properly consider, first, the qualifications of the anesthetiser; second, the apparatus to be employed; third, the preliminary preparations for the patient; and fourth, the technique of administration.

*Qualifications of the Anesthetiser.*—The custom of relegating the administration of chloroform to a student or to the least experienced physician, to nurses and even to the laity, is to be deprecated. So strong are my convictions upon this point, that I am perfectly sincere in saying that were I to require an operation involving the administration of chloroform, I should feel much more solicitous concerning the ability of the anesthetiser than of the surgeon, unless the operation were one requiring exceptional experience and skill. The anesthetiser should be experienced, cool and so fully appreciative of the great responsibility of his position as to give his entire care and attention to his patient. It is a common thing to see the anesthetiser's eyes fixed upon the operation instead of the patient. I have had several most unpleasant experiences, fortunately none of them fatal, from carelessness and inattention to his own work on the part of the anesthetiser.

*The Apparatus.*—This should include a mask (Schimmelbusch's is the one I prefer), chloroform dropper, a mouth-gag, strong tenaculum and sponges upon holders. A hypodermic syringe with digitalis and strychnia should be at hand and ready for instant use. The advantage of the mask is its simplicity and comparative cheapness. A still cheaper and practical substitute can be made from telegraph wire. The gauze covering the frame should, after each operation, either be boiled or thrown away. There is no question but that diphtheria, scarlatina, erysipelas and other contagious diseases have been communicated to healthy persons by means of infected masks, and no person should be exposed to this risk when the prevention is so easy. I have purposely substituted the strong tenaculum for the regular tongue forceps, for the reason that the latter is not an effectual instrument. Furthermore, it inflicts considerable injury to the tongue, which not only causes much discomfort to the patient in the days succeeding operation, but it may in children seriously interfere with their alimentation. The strong tenaculum, fastened into the back part of the tongue, is a less harmful and much more efficient instrument.

*Preliminary Preparations for the Patient.*—The physical condition of every patient about to take chloroform should be as accurately estimated as possible. Especially should the lungs, heart and kidneys be carefully and systematically examined. It is only by so doing that we recognise sources of possible danger. Where time permits, these examinations should be made sometime previous to the administration of the chloroform. It is cruel to begin the examination of the heart just before the patient begins to inhale the anesthetic. His mind, already much disturbed, is further distressed by visions of possible heart failure, and this new mental agitation is added to that already existing, and this condition adds something to the danger without a doubt. The ordinary diseases affecting the organs above alluded to do not contraindicate the use of chloroform (fatty degeneration possibly excepted), but existing diseases should make us doubly watchful, as they unquestionably add to the risk. Strong, healthy adults should take no food for four or five hours previous to operation. In those less vigorous I have for some years allowed a small cup of tea, containing a little brandy, half an hour before the chloroform was given. This rarely induces vomiting and is of distinct value as a stimulant to the circulation. The clothing should be light, but warm and thoroughly loose about the neck, thorax and abdomen. Constriction at these places easily leads to embarrassment of the circulation. The mouth and pharynx should be inspected for foreign materials, such as gum, chewing tobacco, and the like; false teeth and plates are, of course, to be removed. The patient should lie on the back with the head on a level with the remainder of the body at the commencement of anesthesia; and should the operation necessitate the turning of the patient upon the side or abdomen, this may be done after full anesthesia. The lips and nose should be covered with vaseline or some unguent, especially in the case of children. A third person should always be present for obvious reasons. The patient, especially if young or nervous, should not see the instruments and other paraphernalia connected with the operation.

*The Technique of Administration.*—Having covered the mask with some porous material to insure the admixture of plenty of air, we may begin by dropping a few drops upon the mask and holding the same a little distance from the nose and mouth. Beside the discomfort to the patient of beginning with too concentrated vapor, there is positive danger of producing arrest of breathing by the irritation of the terminal fibers of the fifth nerve in the nasal mucous membrane and of the superior laryngeal in the larynx. At most, not more than twenty drops should be put on the mask at one time. These may be lessened as anesthesia proceeds and should not be reapplied until the dark stain, caused by the preceding drops, has passed away. In a short time it will be found that two or three drops at a time suffice to maintain complete surgical anes thesia. Giving chloroform in this way has several advantages:

1. It does not frighten the patient or make him uncomfortable.

2. The excitement stage is almost always absent.

3. After-effects, like nausea and vomiting, are either absent or less severe.

4. The narcosis can be maintained for hours without danger.

5. The amount of chloroform used is less than one-half that in the ordinary way.

6. The danger from an overdose is almost *nil*.

7. The method requires the undivided attention of the anesthetiser, who is thus prevented from watching the operation to the neglect of his patient. The common practice of pouring a quantity of chloroform upon the mask to make it "last," that the anesthetiser may observe the operation with less interruption, is criminal.

The patient should close the eyes and either take full, deep and regular inspirations or, if too nervous to do this, an excellent substitute is to count, a procedure which insures regular breathing. Quietude about the patient, kind encouragement, removing the mask from time to time to give more air, telling the patient some of the sensations he may expect, such as slight choking, and hammering noises in his ears, and that when he begins to swim off into space he must not resist, will make most persons much more tractable. They feel assured that what they are experiencing is the expected and not of bad omen. The explanations and assurances are not trivial, for they aid in obtaining tranquility, which in turn lessens the liability to respiratory and circulatory disturbances. In about five minutes, some sooner and some later, the average person will have become insensible. This period not infrequently, however, varies and no one can ever predict with certainty as to the time that will be required in a given case to produce anesthesia. I recently operated upon a case in which an experienced practitioner had produced not even unconsciousness to the voice in one hour. Squibb's chloroform had been used, and after a time all air was excluded, so far as towels over the mask could shut it out. Investigation showed that the mask had been kept too wet. A dry mask and the drop method of administration produced anesthesia in five minutes.

*Signs of Complete Anesthesia.*—An operation or examination that is attended with great pain should never be begun until complete anesthesia has been obtained. How may we know when we have obtained it? A common practice is

(*a*) *The Cornea.*—To touch the cornea, which ordinarily becomes insensitive at this time. Not infrequently, however, this insensibility long precedes full anesthesia and thus may easily mislead. I have experienced this many times. Too frequent touching obtunds corneal sensitiveness and may also lead us into error. Pricking the inner surface of the thigh is a very good test, as Chassaignac long ago pointed out.

(*b*) *The Pupils.*—The action of the pupils is to be observed, notwithstanding that it is not the same in all persons. The sphincter pupillæ or iridis is governed by the oculo-motor nerve, the dilator by the sympathetic. Usually the pupils behave as follows: at first they dilate; as the narcosis deepens they become contracted and remain so during full anesthesia. As feeling returns they begin to dilate again. However, if in full anesthesia, the pupils suddenly and widely dilate, it is a sign of collapse and calls for the immediate withdrawal of the chloroform. While in many cases we may get reliable information from the condition of the pupils, they are by no means to be implicitly depended upon. For instance, while ordinarily the pupils are contracted in full anesthesia, it not infrequently happens that they remain widely dilated from the beginning to the end of the operation. In fact, one must watch the patient for some time after narcosis has been established and observe the effect of larger and smaller doses of chloroform before correct inferences can be drawn.

(*c*) *The Respiration.*—By far the most important and reliable indicator of normal anesthesia is the respiration. This should be seen or heard throughout the operation and should be of a soft, regular, snoring kind. In the vast majority of cases it is the first function to give the signal of danger, the adherents of ether to the contrary notwithstanding. As a source of information the pulse is not to be compared with it, for the pulse may vary greatly from innocent causes and give the anesthetiser and operator much needless alarm. During a difficult operation not long ago, in which a careful dissection was necessary and which did not permit undue haste, my anesthetiser gave me the cheering information that the patient had no radial pulse. A glance at the lips showed them to be of good color and further investigation revealed the absence of pulse to be due to a strained position of the arms above the head. Again, the pulse always becomes markedly weakened just before emesis and thus may needlessly alarm us.

(*d*) *The Color of the Face.*—The color is usually a reliable index of the patient's general condition. The appearance of cyanosis or of pallor demands immediate attention. Cyanosis means embarrassed respiration. Pallor indicates circulation depressed from surgical shock, insufficient breathing (asphyxial syncope) or an overdose of chloroform. It may also, and often does, precede vomiting, but when so caused need not occasion alarm.

The operation ended, the patient should not be quickly or rudely awakened from his sleep. It is oftentimes hard to prevent relatives and over-anxious friends from trying to elicit signs of consciousness. It is much better, however, to let the patient awaken naturally, as they have less nausea and vomiting and feel better generally when left alone. This is especially true of children. The anesthetiser should remain by his patient until consciousness has returned. By so doing such accidents as choking from vomit, hemorrhage, and the like, will not be serious if they occur at all. The room should be kept dark and quiet, but well ventilated. The patient should be given food as soon as the appetite returns.

For nausea and vomiting, hot water, with or without sodium bicarbonate, in half teaspoonful doses, iced champagne or cerium oxalate, may be used. A hot application to the epigastrium is often also very serviceable.

Before discussing the accidents and dangers which may occur during chloroform narcosis, it is fitting to consider how chloroform kills. There is a common impression among the profession that it invariably exercises its lethal effect upon the heart. That it sometimes does so there can be no doubt, but that this is its usual action is certainly not so. Its primary effect, in the vast majority of cases, is upon the respiration, popular belief notwithstanding. The reason for the impression regarding the action of chloroform on the heart is probably to be found in the varying opinions of experimenters, therapeutists and surgeons, which one finds at every turn. However, a careful investigation of statistics will conclusively show that after its effect, primarily upon the vasomotor system, the chief action of chloroform is upon the respiratory centers in the medulla. This is the experience, in the main, of both experimenters and practical surgeons. However, as before said, chloroform may kill by acting directly upon the heart muscle. The sphygmograph usually shows true depression of the heart, and the pulse almost invariably becomes more feeble under its use, owing no doubt to cardiac dilatation. It also causes marked vasomotor dilatation, which condition greatly influences respiration and the action of the heart. The common phenomenon of gasping respiration in ordinary faintness is probably due to sudden vascular dilatation, rather than to any direct failure of the heart. So, also, the exceedingly rapid pulse of shock occurs with the relaxed blood-vessels found in this condition. From what has been said we may easily appreciate that those diseases which affect the respiratory or circulatory systems add an element of danger when patients so afflicted require chloroform anesthesia. As Hare tersely and clearly puts it:

> Supposing that the amount of depression from very full doses of chloroform equals twenty-five units, this amounts to little in the normal heart; but if the heart be depressed twenty-five additional units by disease, the depression of fifty units may be fatal, particularly if to this fifty is added twenty-five units more of depression through fright and cardiac engorgement, through disordered respiration or struggling.

Among these diseases may be mentioned fatty degeneration of the heart, valvular diseases, atheromatous degeneration of the walls of the vessels and particularly of the coronary arteries, anemia, chronic pulmonary disease, Bright's disease and chronic alcoholism. Fatty degeneration of the heart muscle is especially dangerous, but whenever any of the above-named diseases exist, singly or in combination, chloroform must be given with the greatest caution, and especially so whenever respiratory or circulatory disturbances have been brought about by these diseases. The subject of the influence of pathological conditions over death from chloroform is a most interesting and important one, but time does not permit any further discussion of it on this occasion.

Anxiety and fright, while not in a sense pathological conditions, certainly are depressants of the heart and may, in one way or another, produce dangerous or fatal symptoms, as has been shown in cases where death has occurred before any chloroform has been administered. For instance, Desault was about to perform a lithotomy, and to demonstrate the line of incision he drew his finger nail over the patient's perineum. The latter gave a loud cry and instantly died. Cazenave, who was about to operate upon a very nervous patient, did not give chloroform, but made a pretense by putting a cloth over the patient's nose. The respiration and heart stopped, and the patient was dead. Simpson's first patient died in a similar way. The chloroform bottle was accidentally broken and the chloroform spilled. The operation, a herniotomy, was begun without the anesthetic. On incising the skin the patient died. It is easy to see how, oftentimes, chloroform may get credit for causing death which it does not deserve.

Impure chloroform has already been alluded to as dangerous. Here again we must be cautious in ascertaining the true cause of a fatal effect. Just how often impure chloroform leads to disaster it is impossible to say, but it seems not unlikely that it is more frequent than is commonly supposed.

Let us now consider some of the accidents and dangers which may appear during chloroform narcosis.

1. *Respiratory Embarrassment or Failure.*—This may be due to (*a*) mechanical obstruction, (*b*) paralysis of respiration and (*c*) spasm of the respiratory muscles.

(*a*) Mechanical obstruction may be due to various causes. The lips of edentulous subjects may become approximated and prevent inspiration. Lenhart reports a case of asphyxia in a girl with a very pointed nose and exceedingly thin alæ nasi, which through atmospheric pressure were brought tightly against the septum, thus closing the anterior nares. The tongue may become engorged, it may be drawn back by muscular spasm, or fall against the pharyngeal wall from paralysis. Intranasal conditions, diminishing the caliber of the nares, partially completed deglutition, laryngeal spasm or foreign bodies in the same, such as blood, vomit, mucus, and the like, are among the sources of obstruction.

(*b*) Paralysis of respiration is usually caused by an overdose of chloroform, and when so caused is accompanied with symptoms of collapse—namely, pallor, a very feeble pulse, and the like. It may, however, depend upon syncope or pulmonary disease.

(*c*) Spasm of the respiratory muscles is not uncommon. It is usually not severe, but may cause death. It occurs just before anesthesia has become complete, and its chief feature in the beginning is the extreme rigidity of the chest which, in adults, it may be impossible to overcome.

2. *Circulatory Disturbances or Failure.*—As has been previously said, these disturbances are almost invariably connected with, or dependent upon, respiratory embarrassment or failure. This is most important to remember and will be alluded to again when considering the treatment to be pursued in cases of accident. The circulation may be depressed by (*a*) surgical shock, (*b*) syncope following embarrassed or arrested respiration (asphyxial syncope), (*c*) an overdose of chloroform and (*d*) nausea with or without vomiting.

(*a*) Surgical shock varies according to the nature and severity of the operation and the resistance of the patient. It is usually gradual in its appearance, but when great irritation of the vagus or solar plexus has been caused during operation, it may appear most suddenly and cannot then be differentiated from syncope due to too much chloroform. The symptoms are classic and need not be enumerated.

(*b*) Syncope following embarrassed or arrested respiration. The first symptom of this condition is cyanosis, which gradually or rapidly gives place to pallor and cardiac failure. It may occur alike in the feeble or the vigorous. In the latter the asphyxia begins when the patient resists the chloroform. The way in which it occurs is as follows: the patient struggles, then holds his breath; the lungs contain chloroform, which the blood is absorbing while the patient is not breathing; the right side of the heart becomes fuller, all the cardiac cavities become distended, the blood pressure falls and finally cardiac action ceases.

(*c*) An overdose of chloroform causes fatal depression of the circulation and of the heart. The face becomes ashy, the eyes staring and expressionless, and finally the respiration ceases almost coincidently with the circulation.

(*d*) Nausea, with or without vomiting, sometimes causes death from too great a fall in the blood pressure in feeble or diseased patients. In these cases the suspension of breathing incident to vomiting may suffice to induce asphyxia followed by syncope, ending in death.

Inasmuch as any or many of these conditions may arise at any stage of chloroform narcosis, what can we do to remove them speedily and safely? We may divide our therapeutic measures into (*a*) preventive and (*b*) immediate.

(*a*) *Preventive Measures.*—Primary syncope is usually due to the irritating effect of the chloroform vapor, upon the terminal branches of the fifth nerve in the nasal mucous membrane and of the superior laryngeal in the upper part of the larynx. These run to the central nervous system, and reflexes originating there manifest themselves along the motor tracts leading to the muscles governing respiration and through the vagus, which governs the heart. There are various ways of rendering these reflexes more or less harmless.

Rosenberg, in the last congress of surgeons in Germany, drew attention to the fact that by cocainising the nasal mucous membrane and that of the pharynx and larynx before administering chloroform, we might abolish the reflexes called forth by the irritation. He found by experiment that at the beginning of anesthesia, (the blood pressure equaling 100,) the systole represents 210 and the diastole 40. Under normal conditions, the blood pressure being the same, the systole equals 110 and the diastole 90. The difference is due to the irritation of the terminal fibers of the fifth nerve in the nasal mucous membranes, which in turn produced respiratory disturbances. To cocainise the nares with a 2 per cent. solution, either with a camel's hair brush or a spray, is good practice, especially in the case of patients whose condition, for one reason or another, requires that the extra precautions be taken. Patients thus treated struggle less and come under the anesthetic more quickly. If no cocaine is at hand, we may force the patient to breathe through the mouth by holding the nose, as practised by Guerin. It may be well, just at this point, to repeat the admonition never to exhibit chloroform vapor in too concentrated form. The procedure is extremely dangerous and, therefore, always to be avoided.

(*b*) The use of atropine previous to anesthesia in part removes the danger arising from excessive irritation of the vagus. Hare believes

That atropine enables more chloroform to be given without circulatory depression than can be used if no atropine be administered, and that there is good reason to believe that the use of atropine by surgeons, for the purpose of stimulating the respiratory functions or preventing cardiac inhibition by irritation of the vagus, in reality prevents dangerous symptoms chiefly by its vasomotor influence.

(*c*) Morphine in one-eighth to one-fourth grain doses, given fifteen minutes before the beginning of anesthesia, renders the brain much less impressionable, and, therefore, insures more tranquillity on the part of the patient. I am quite sure, however, that in some cases morphia favors respiratory syncope, and, therefore, it should be given in selected cases only and not as a routine practice, as is so commonly done in hospitals by many surgeons. When properly used it is of distinct value not only in rendering the patient more calm, but also in curtailing the time and amount of chloroform required. It is usually given in combination with atropine and in this way the combined effects of both drugs may be secured.

(*d*) Digitalis should be used in certain cases before anesthesia, according to H. C. Wood. He finds that digitalis markedly increases arterial pressure and has seen death apparently averted by its use. In one or two experiments, in which large amounts had been used, he saw sudden systolic cardiac arrest, showing that digitalis in sufficient dose could overcome the effects of chloroform. He says that "in all cases of weak heart in man a full dose of digitalis, given hypodermatically before the administration of chloroform, would greatly lessen the danger of cardiac collapse."

So much for the preliminary or preventive treatment of chloroform accidents. Given a case, however, in which the treatment has been inoperative in preventing trouble and we are brought face to face with impending death, what shall we do?

(*b*) *Immediate Treatment.*—Shall we stop to debate whether we have to deal with respiratory or circulatory failure, and as to what means we should employ in each kind of case? If we do, we shall often, perhaps always, allow our patient to die. One thing we do know from experience, and that is that artificial respiration exercises a great influence upon the circulation as well as the respiration. During inspiration a strong suction action is effected upon the thin-walled great veins, the right auricle becomes filled, and the pressure exercised by expiration aids in the emptying of the blood through the auriculo-ventricular opening into the right ventricle. On the other hand, a measure which will cause the paralysed heart to contract will have no effect upon arrested respiration, unless the blood being oxygenated in the lungs produces irritation, and hence we have in artificial respiration an agency which stimulates both respiration and circulation. In fact, experience is constantly proving that in this procedure we have a life-saving measure of the greatest importance and one which should be invariably employed. Bearing these considerations in mind, how shall we act in a case where death is threatening?

1. We must clear the upper air-passages of any foreign substances which may be producing mechanical obstruction. At the same time, we remove the pressure of paralysed parts upon the larynx by carrying out the following rule laid down by Hare:

> Place the index finger of each hand upon the corresponding cornua of the hyoid bone, whilst the middle fingers rest upon the angle of the jaw, and then press forward and upward, the same force serving to extend the head upon the neck; if this fails to open the glottis, by means of a tenaculum, thrust far back into the base of the tongue, draw it forward.

It will at once be seen how radically this procedure differs from Howard's, which is the one usually described and recommended in text-books. There can be no doubt that Howard's method does not prevent but even favors the approximation of the tongue and soft palate, which thus cuts off the entrance of air through the mouth. Inasmuch as it is the tongue, and not the epiglottis, which usually causes obstruction, it is easy to understand that Howard's plan must often prove inefficacious. Mechanical obstruction, if existing, having been thus dealt with, we should immediately begin with artificial respiration.

2. *Artificial Respiration.*—The chest should first be compressed, to expel any chloroform vapor remaining in the lungs. The simplest and best method, in my judgment, of performing artificial respiration is Sylvester's, which has for its object the use of the arms as levers, acting so as to expand the chest-walls by means of the muscles placed between the limbs and the trunk, the origins of the muscles acting now as insertions and *vice versa.* This should be maintained for anywhere from one-half to one and one-half hours, or even longer when any hope is offered of ultimate success. I have seen more than one life saved, I am sure, by persistence and energy, and where a human life is at stake no effort is too great. If artificial respiration is ineffectual, as for instance in spasm of the respiratory muscles with rigid chest, direct inflation of the lungs with bellows may be employed with success. The Marshall Hall method of artificial respiration has many adherents. It is, however, not so efficient as the Sylvester method, which pumps into the lungs a much larger volume of air.

After employing artificial respiration for two or three minutes, in the manner just described, should it be attended with increasing signs of syncope, we must immediately resort to (*c*) Nelaton's inversion method, which consists in suspending the patient by the lower limbs, head downward, and continuing the respiration by placing one hand on the back and the other on the sternum. During the last winter I saved the life of a patient by this method. Just how it acts is uncertain, but there can be no question of its value. Under this treatment the pulse and respiration usually immediately improve. The restoration to a horizontal position should be very gradual, as not infrequently a sudden change from the vertical to the recumbent is attended with a renewal of the syncope.

(*d*) *Rhythmic Compression of the Heart.*—This is another procedure of unquestioned value, which has the great advantage that it can be used coincidently with artificial respiration. As recommended and described by König, it consists of short, powerful impulses, with the fingers over the precordium, at the rate of about 120 per minute. In a case of König's, with each impulse a similar one could be felt in the radial pulse. The patient recovered. While the method of compressing the heart for this purpose is not new, the method of König is peculiar, in that the number of impulses given corresponds with the number of heart beats in the individual.

(*e*) *Electricity.*—What is the value of this agent in the class of cases under consideration and how may it be used ? It may be applied to the phrenic nerve to stimulate inspiration, one electrode being placed over the nerve on the right side and the other over the sternum, or it may be used as a galvanic needle, which is thrust into the heart substance, or it may be employed to stimulate the nasal mucous membrane. When we remember that the phrenic nerve governs inspiration only and that expiration is just as essential in this class of cases, that a strong faradic current is a veritable poison to the heart, quickly causing paralysis in diastole, and that the use of the galvanic needle is too dangerous to be recommended, we must conclude that as an excitant of respiration electricity is inefficient and as a stimulant to the heart is dangerous. Fortunately a battery is rarely at hand and, therefore, only infrequently does harm.

(*f*) *Drugs.*—May we expect assistance from drugs ; and if so, which shall we employ ?

Alcohol is very commonly used in one form or another as a stimulant in cardiac depression. It is bad practice. In fact, it ought never to be used in chloroform syncope, for the reason that instead of antagonizing it only intensifies the effect of the anesthetic and lessens the dose necessary to produce death. In large doses it causes a fall in arterial pressure and a smaller pulse. Wood even goes so far as to say "that in not a few cases deaths which have been attributed to ether and other anesthetics have been in fact due to the alcohol which has been given the patient," and he further observes "that it should be an unalterable rule of practice that no alcohol should ever be given to the patient suffering from anesthetic cardiac failure." What applies to alcohol is applicable with equal force to most of the so-called cardiac stimulants.

In a general way we may say of them that they are not useful, often harmful, and time spent in using them can be employed to better advantage.

Digitalis has already been mentioned as a true antagonist of chloroform and should be used in cases requiring increase of cardiac force. It is best given hypodermatically in 5 to 10 drop doses.

Amyl nitrite is a drug much used in chloroform syncope. My own experience with the drug has been quite extensive. I have used it in solution (3 or 4 drops to the ounce of chloroform) and alone. The only death from chloroform I have ever had occurred with this mixture. In late years I have used only pure chloroform. The drug is dangerous, without doubt, if too much be given, and the ordinary dose is without effect usually. It does not seem to me a drug which deserves the reputation it enjoys.

Strychnia, in doses of 1-20 of a grain, raises arterial tension and deepens the respirations. It is, therefore, a drug which antagonises chloroform and can be safely and advantageously employed.

*Cocaine.*—This is also a powerful respiratory stimulant. Wood found that, in a chloralized dog, when the respirations had been raised as far as was safe with strychnia, cocaine was able still further to improve them, and he suggests that a combination of strychnia and cocaine would act more efficiently in the accidents of anesthesia than would either alkaloid by itself.

The injection of these stimulants directly into the heart muscle would seem to be justifiable. Dr. L. Pierce Clark (*Boston Medical and Surgical Journal*, April 18, 1895,) reports three cases of delirium tremens treated with strychnia injected into the heart substance. One case received 1-15 of a grain, followed in one hour with 1-20 grain, and recovered in five days. A second case required but one injection. I have had no personal experience with this method, but see no objection to it. How much the effect is due to mechanical irritation and how much to the action of the drug proper it is impossible to say, but probably both factors have their influence.

519 Franklin Street.

---

Fractures.—The object of the surgeon in treating fractures about the joints should be: (1) To allow free circulation in the limb; (2) to obtain complete rest for the injured structures until they assume their normal condition; (3) to posture so that the callus exudation shall not unduly hamper the joint movements.—*Medical Record.*

